# Coordinated evolution of co-expressed gene clusters in the *Drosophila *transcriptome

**DOI:** 10.1186/1471-2148-8-2

**Published:** 2008-01-07

**Authors:** Jason G Mezey, Sergey V Nuzhdin, Fangfei Ye, Corbin D Jones

**Affiliations:** 1 Department of Biological Statistics and Computational Biology, Cornell University, Ithaca, NY 14853, USA; 2 Molecular and Computational Biology, University of Southern California, Los Angeles, CA 90089-2910, USA; 3 Department of Biology and Carolina Center for the Genome Sciences, University of North Carolina at Chapel Hill, Chapel Hill, NC 27599-3280, USA

## Abstract

**Background:**

Co-expression of genes that physically cluster together is a common characteristic of eukaryotic transcriptomes. This organization of transcriptomes suggests that coordinated evolution of gene expression for clustered genes may also be common. Clusters where expression evolution of each gene is not independent of their neighbors are important units for understanding transcriptome evolution.

**Results:**

We used a common microarray platform to measure gene expression in seven closely related species in the *Drosophila melanogaster *subgroup, accounting for confounding effects of sequence divergence. To summarize the correlation structure among genes in a chromosomal region, we analyzed the fraction of variation along the first principal component of the correlation matrix. We analyzed the correlation for blocks of consecutive genes to assess patterns of correlation that may be manifest at different scales of coordinated expression. We find that expression of physically clustered genes does evolve in a coordinated manner in many locations throughout the genome. Our analysis shows that relatively few of these clusters are near heterochromatin regions and that these clusters tend to be over-dispersed relative to the rest of the genome. This suggests that these clusters are not the byproduct of local gene clustering. We also analyzed the pattern of co-expression among neighboring genes within a single *Drosophila *species: *D. simulans*. For the co-expression clusters identified within this species, we find an under-representation of genes displaying a signature of recurrent adaptive amino acid evolution consistent with previous findings. However, clusters displaying co-evolution of expression among species are enriched for adaptively evolving genes. This finding points to a tie between adaptive sequence evolution and evolution of the transcriptome.

**Conclusion:**

Our results demonstrate that co-evolution of expression in gene clusters is relatively common among species in the *D. melanogaster *subgroup. We consider the possibility that local regulation of expression in gene clusters may drive the connection between adaptive sequence and coordinated gene expression evolution.

## Background

The non-random arrangement of genes in the genome is intimately connected to the pattern of gene expression across the genome [1]. While the connection between gene location and expression has been known for some time in prokaryotes [2], a similar genome-wide connection between gene order and gene expression has relatively recently been identified in eukaryotes [3]. Clusters of physically adjacent genes that are co-expressed are now known to be common in eukaryotic genomes and have been reported in yeast [4,5], plants [6], worms [7-10], fruit flies [11-13], mice [14-16], and humans [16-20].

A number of mechanisms have been proposed to explain the existence of these co-expression clusters including the presence of duplicate genes that are in close physical proximity, shared regulatory regions, chromatin-level regulation, and common pathway or tissue regulated expression of physically clustered genes [1,8,21]. Similarly, a number of hypotheses have been proposed concerning the interplay between transcriptome evolution and genome organization that can explain the existence of co-expression clusters including positive selection for genomic rearrangements leading to close physical proximity of co-expressed genes [22] and purifying selection against genomic rearrangments that break-up co-expression clusters [5,18]. The possibility that co-expression results in correlated rates of sequence evolution among cluster genes has also been proposed [23] and a recent analysis has found evidence of co-evolution of tissue specific expression of adjacent genes [24]. Although not fully resolved, these analyses have led to a clearer picture of both the pattern of co-expression clusters within species and explanations concerning why gene expression is often coordinated among physically adjacent genes.

If there is a physical clustering of coordinated gene expression within species, then it is likely that gene expression can also *evolve *in a coordinated manner [24]. A block of consecutive genes where expression evolves in a coordinated manner will leave an evolutionary signature that can be detected by non-zero expression correlation among neighboring genes when analyzing multiple species. Therefore, just as correlated expression profiles are used to identify co-expression among genes within species [4,12] the same approaches can be used to analyze co-evolution of expression in gene clusters when comparing gene expression among species. Instead of analyzing the correlations in gene expression among developmental stages, environments, tissue localizations, etc. [1], we consider the correlations among mean gene expression levels estimated for each species. This approach will identify clusters of genes where the evolution of expression is *not *a gene independent process.

**Figure 1:**
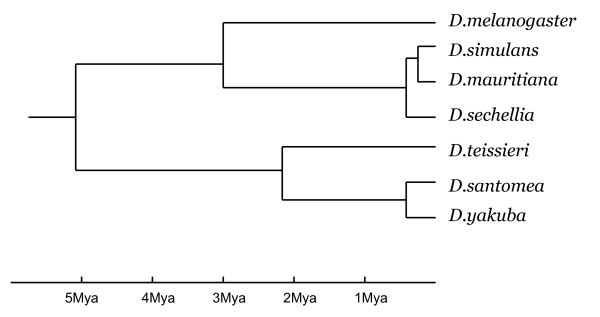
Relationships among the seven species in the *D. melanogaster *subgroup that were analyzed. Times of speciation events follow estimates from [26].

Our goal is to identify clusters of genes that show consistent patterns of coordinated expression evolution among species of *Drosophila*. We assayed genome-wide gene expression levels using Affymetrix GeneChip Arrays in three-day-old male adults under standardized environmental conditions [25]. The following seven species in the *D. melanogaster *subgroup were analyzed: *D. melanogaster*, *D. simulans*, *D. sechellia*, *D. mauritiana*, *D. santomea*, *D. teissieri*, and *D. yakuba*. The relationships among these species are well established (Fig. 1) and represents a taxonomic sampling that spans ~6 million years [26]. Clusters identified for these species are therefore of value for understanding how the transcriptomes of species evolve across time scales on the order of one-hundred thousand to several million years.

A number of methods have been applied to the identification of co-expression clusters within species using microarray expression data [4,12,23,27-29]. The most common of these is calculation of a statistic based on the estimated correlation matrix for blocks of consecutive genes, generally the mean of N*(*N*-1)/2 correlations when considering *N *consecutive genes [12]. For the current study, we use a different statistic to summarize the correlations among *N *genes: the fraction of variation explained by the leading eigenvalue of the correlation matrix. This statistic describes the maximum fraction of variation that can be explained by a linear function of the original variables after scaling the variance of each variable to one. This statistic therefore provides an intuitive description of the degree to which a set of genes act as a single unit because the closer this ratio is to "1" the greater the degree that expression of the genes are completely correlated, regardless of whether the correlations among any gene pair are positive or negative. While this statistic has not been explicitly applied to the analysis of co-expression, the leading eigenvalue(s) of a correlation or covariance matrix are commonly used to summarize the structure of correlated variation [30,31].

Our analysis shows that a considerable proportion of transcriptome evolution among species in the *D. melanogaster *subgroup occurs via co-evolution of expression in clustered genes. Comparison of the locations of clusters that reflect coordinated evolution of gene expression across taxa to clusters of coordinated expression within the species *D. simulans *demonstrated a lack of correspondence in locations. This implies that different mechanisms may be responsible for producing co-expression clusters within species and those producing co-expression clusters that evolve in a coordinated manner. We additionally analyze a number of genome organization, functional, and evolutionary aspects to identify over-(under-) representation with clusters displaying coordinated expression within *D. simulans *or coordinated expression evolution among the seven species. Of these, the most interesting are genes that show a signature of adaptive evolution in their coding sequences. A previous analysis of tissue co-expression within mice and humans did not find a significant positive correlation between rates of non-synonymous substitutions (*K*_*A*_) and co-expressed genes [23], although the ~75 million years since humans and mice diverged likely limited the power of this analysis [32]. Here, we find that co-expression clusters that vary *within D. simulans *are not enriched for adaptive evolving loci. However, genes with an adaptive evolutionary signature are over-represented in clusters where expression is co-evolving *among *species. This result points to a connection between coordinated gene expression evolution and adaptive evolution in coding regions of genes, although the exact nature of this connection is still unknown.

## Results and Discussion

**Figure 2:**
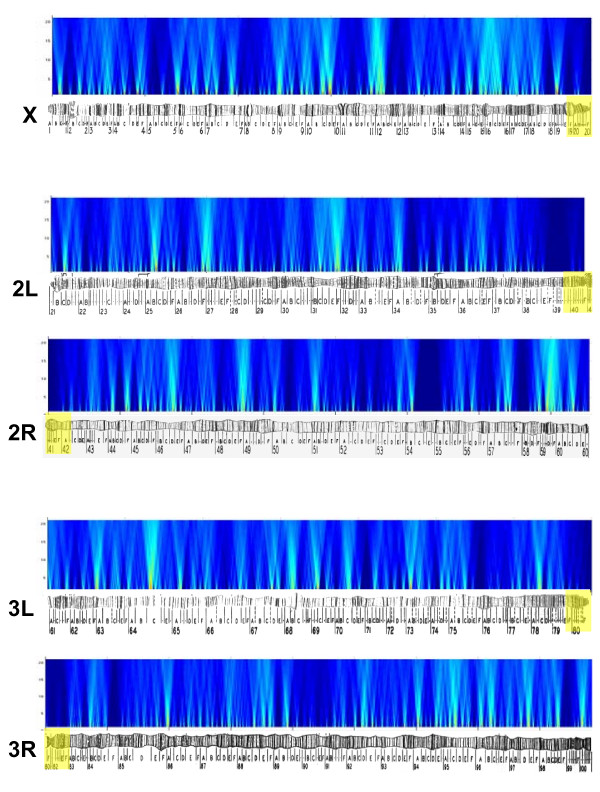
Sliding window heat map of p-values resulting from the clustering analysis across species projected onto the *D. melanogaster *genome. Approximate position on chromosomes is plotted along the x-axis and window size on the y-axis. Centromere proximal regions are indicated by yellow shading on the chromosome. The spectrum runs from highly significant p-values (red) to highly non-significant p-values (dark blue).

Clusters of genes evince coordinated evolution of expression across the seven species (Figure 2). On all chromosomes at all scales, there were far more windows with significant coordinated expression evolution than expected at random (Table 1). Many of these significant windows were identified across multiple window sizes and likely reflect a single larger block of genes where expression evolution is coordinated [12] (we present combined significant windows at different cutoffs in Additional files 1, 2, 3, 4, 5, 6, 7, 8, 9, 10). These windows were also robust to the removal of individual species and therefore were not being driven by evolution in a specific lineage (results presented in Additional files 1, 2, 3, 4, 5, 6, 7, 8, 9, 10). In addition, there was no detectable difference between the absolute level of transcript abundance as measured by the arrays for neighboring genes where expression displays co-evolution compared to other groups of neighboring genes (p-values > 0.05 for all window sizes). Because we used the *D. melanogaster *as the reference genome for ordering genes along chromosomes there is the possibility that genome re-arrangements would lead to some of these significant clusters to include non-physically adjacent genes in some of the species. Our results are, however, robust to this issue as we found that breakpoints between *D. simulans*-*D. melanogaster *or *D. yakuba*-*D. melanogaster *interrupted clusters where expression is co-evolving no more frequently than expected by chance, which is consistent with a previous analysis of co-expression within species [33].

**Table 1:** Number of significant windows where expression is co-evolving.

Chr	Pval	2	4	6	8	10	12	14	16	18	20
**X**	0.05	31(28.95)	82(75.8)	95(96.75)	114(105.35)	116(109.45)	123(111.2)	117(112.3)	121(112.9)	113(113.25)	126(113.5)
**X**	0.01	13(5.79)	22(15.16)	25(19.35)	22(21.07)	30(21.89)	28(22.24)	30(22.46)	33(22.58)	30(22.65)	33(22.7)
**X**	0.001	5(0.579)	7(1.516)	9(1.935)	9(2.107)	9(2.189)	9(2.224)	12(2.246)	10(2.258)	7(2.265)	5(2.27)
**2L**	0.05	37(31.8)	79(82)	110(106.1)	121(118.3)	135(123.95)	132(126.15)	143(127.3)	139(128.35)	144(129.05)	137(129.35)
**2L**	0.01	9(6.36)	19(16.4)	26(21.22)	27(23.66)	29(24.79)	33(25.23)	40(25.46)	26(25.67)	35(25.81)	39(25.87)
**2L**	0.001	2(0.636)	9(1.64)	5(2.122)	5(2.366)	4(2.479)	7(2.523)	15(2.546)	10(2.567)	10(2.581)	8(2.587)
**2R**	0.05	47(37.9)	113(94.9)	151(121.55)	163(133.4)	150(138.7)	139(140.95)	126(142.15)	143(143.05)	143(143.6)	170(144.15)
**2R**	0.01	11(7.58)	24(18.98)	39(24.31)	45(26.68)	48(27.74)	48(28.19)	57(28.43)	63(28.61)	65(28.72)	68(28.83)
**2R**	0.001	3(0.758)	10(1.898)	16(2.431)	26(2.668)	24(2.774)	31(2.819)	30(2.843)	41(2.861)	34(2.872)	41(2.883)
**3L**	0.05	46(38.25)	105(94.8)	133(118)	129(128.4)	145(132.95)	141(135.1)	147(136.3)	147(137.1)	135(137.65)	133(138)
**3L**	0.01	16(7.65)	28(18.96)	38(23.6)	33(25.68)	34(26.59)	39(27.02)	39(27.26)	33(27.42)	25(27.53)	19(27.6)
**3L**	0.001	9(0.765)	10(1.896)	10(2.36)	10(2.568)	13(2.659)	14(2.702)	5(2.726)	12(2.742)	5(2.753)	1(2.76)
**3R**	0.05	68(50.45)	129(120.65)	148(152.05)	170(165.25)	193(170.9)	198(173.7)	175(175.25)	160(175.8)	161(176)	181(176.05)
**3R**	0.01	15(10.09)	34(24.13)	33(30.41)	39(33.05)	44(34.18)	38(34.74)	37(35.05)	31(35.16)	32(35.2)	27(35.21)
**3R**	0.001	6(1.009)	8(2.413)	12(3.041)	16(3.305)	15(3.418)	11(3.474)	13(3.505)	16(3.516)	6(3.52)	10(3.521)
**4**	0.05	1(0.55)	2(2)	4(2.95)	8(3.6)	8(3.85)	9(3.95)	9(3.9)	5(3.8)	3(3.7)	2(3.6)
**4**	0.01	0(0.11)	2(0.4)	3(0.59)	5(0.72)	6(0.77)	5(0.79)	3(0.78)	5(0.76)	0(0.74)	0(0.72)
**4**	0.001	0(0.011)	1(0.04)	3(0.059)	5(0.072)	6(0.077)	5(0.079)	0(0.078)	0(0.076)	0(0.074)	0(0.072)

**TOTAL**	0.05	230(187.9)	510(470.15)	641(597.4)	705(654.3)	747(679.8)	742(691.05)	717(697.2)	715(701)	699(703.25)	749(704.65)
**TOTAL**	0.01	64(37.58)	129(94.03)	164(119.48)	171(130.86)	191(135.96)	191(138.21)	206(139.44)	191(140.2)	187(140.65)	186(140.93)
**TOTAL**	0.001	25(3.758)	45(9.403)	55(11.948)	71(13.086)	71(13.596)	77(13.821)	75(13.944)	89(14.02)	62(14.065)	65(14.093)

The total number of significant tests obtained from the sliding window analysis for even numbered window sizes are presented with the expected numbers assuming a null distribution in parentheses.

The use of a sliding window approach to identify co-evolution of expression in gene clusters means that tests at a given scale will be correlated with neighboring windows and these tests will also be correlated across window sizes. There is not a clear optimal approach for dealing with the multiple testing problem and the properties of strategies such as estimation of False Discovery Rates (FDRs) [34,35] are not clear in such cases. To assess whether there was a clear genome-wide tendency for coordinated expression evolution, we therefore used a permutation approach using total number of significant tests at a given window size (2, 5, 10, and 20) as a test statistic to assess the null hypothesis that there are no more gene clusters where expression is co-evolving than we would expect at random [12]. With only seven species (samples) these tests are not expected to be particularly powerful. However, the test was still rejected at a window size of 2 (p-value < 0.02) and at a window size of 10 (p-value < 0.04) (although not for window sizes of 5 and 20) indicating that at least on genomic scales spanning 2 to 10 genes, there is genome level co-evolution of gene expression in neighboring genes.

**Figure 3:**
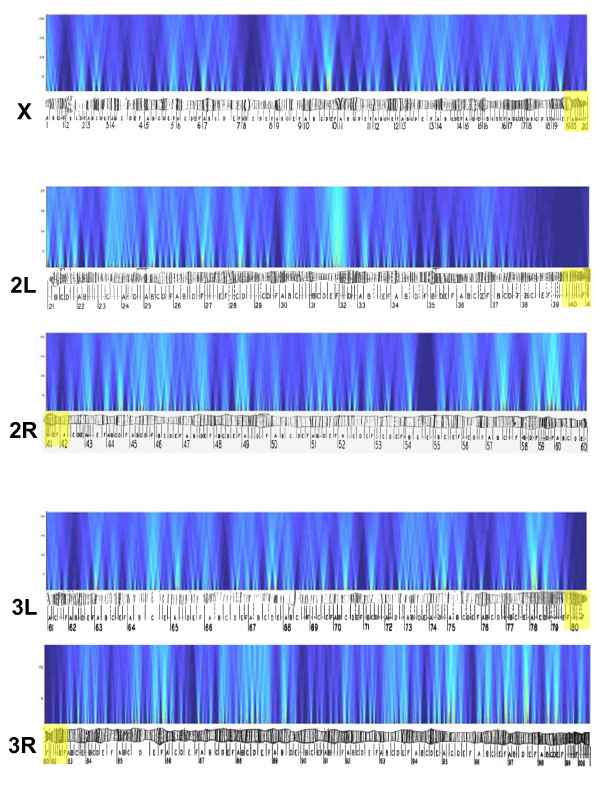
Heat map of p-values resulting from the sliding window analysis within *D. simulans *projected onto the *D. melanogaster *genome. Color coding follows Figure 2.

Similar results were obtained for the analysis of within species co-expression for *D. simulans *(Table 2, Figure 3). Many windows on all chromosomes at all scales were significant. Interestingly, the test of a genome-wide pattern produced significant results for window sizes of 2 (p-value < 0.04) and 10 (p-value < 0.01). While this could be interpreted as an artifact of microaray design [36,37], there is no regular spacing to the distribution of significant windows [28]. Interestingly, there was little overlap between the significant windows identified as evolving across species and being co-expressed within *D. simulans *(Table 3). The number of overlapping windows obtained when comparing repeated analysis of mean expression levels for all species and the number of overlapping windows for repeated analysis of the *D. simulans *data (i.e. non-overlap due to permutation effects) are presented for comparison. Given that many of the co-evolving expression clusters and the co-expression clusters identified within *D. simulans *may reflect false positives, a small fraction of overlap between these cluster types might be expected. However, even at a conservative cutoff (p-value < 0.001) the absolute number of overlapping clusters is still very low (Table 3) indicating that there is little correspondence. It therefore appears that completely different sets of genes are involved in the pattern of co-expression within species compared to those where expression evolves in a coordinated manner across species.

**Table 2:** Number of significant co-expression windows in *D. simulans*.

Chr	Pval	2	4	6	8	10	12	14	16	18	20
**X**	0.05	39(28.95)	93(75.8)	121(96.75)	144(105.35)	138(109.45)	146(111.2)	125(112.3)	129(112.9)	137(113.25)	143(113.5)
**X**	0.01	13(5.79)	28(15.16)	36(19.35)	46(21.07)	41(21.89)	41(22.24)	58(22.46)	48(22.58)	50(22.65)	33(22.7)
**X**	0.001	5(0.579)	9(1.516)	11(1.935)	16(2.107)	24(2.189)	23(2.224)	16(2.246)	13(2.258)	15(2.265)	13(2.27)
**2L**	0.05	31(31.8)	100(82)	142(106.1)	161(118.3)	173(123.95)	170(126.15)	165(127.3)	176(128.35)	186(129.05)	178(129.35)
**2L**	0.01	6(6.36)	29(16.4)	41(21.22)	41(23.66)	45(24.79)	56(25.23)	41(25.46)	52(25.67)	55(25.81)	51(25.87)
**2L**	0.001	4(0.636)	13(1.64)	15(2.122)	11(2.366)	12(2.479)	23(2.523)	22(2.546)	17(2.567)	19(2.581)	22(2.587)
**2R**	0.05	38(37.9)	102(94.9)	141(121.55)	165(133.4)	175(138.7)	187(140.95)	154(142.15)	147(143.05)	147(143.6)	127(144.15)
**2R**	0.01	9(7.58)	27(18.98)	45(24.31)	56(26.68)	60(27.74)	45(28.19)	39(28.43)	47(28.61)	49(28.72)	46(28.83)
**2R**	0.001	3(0.758)	3(1.898)	17(2.431)	18(2.668)	16(2.774)	14(2.819)	9(2.843)	12(2.861)	11(2.872)	11(2.883)
**3L**	0.05	43(38.25)	104(94.8)	129(118)	140(128.4)	143(132.95)	134(135.1)	138(136.3)	146(137.1)	153(137.65)	156(138)
**3L**	0.01	11(7.65)	29(18.96)	33(23.6)	38(25.68)	44(26.59)	36(27.02)	46(27.26)	49(27.42)	60(27.53)	51(27.6)
**3L**	0.001	6(0.765)	14(1.896)	14(2.36)	13(2.568)	17(2.659)	26(2.702)	29(2.726)	28(2.742)	31(2.753)	26(2.76)
**3R**	0.05	55(50.45)	110(120.65)	151(152.05)	168(165.25)	152(170.9)	152(173.7)	159(175.25)	157(175.8)	178(176)	173(176.05)
**3R**	0.01	10(10.09)	27(24.13)	31(30.41)	39(33.05)	35(34.18)	36(34.74)	46(35.05)	44(35.16)	44(35.2)	45(35.21)
**3R**	0.001	4(1.009)	5(2.413)	14(3.041)	9(3.305)	9(3.418)	15(3.474)	14(3.505)	13(3.516)	7(3.52)	10(3.521)
**4**	0.05	0(0.55)	1(2)	0(2.95)	1(3.6)	3(3.85)	3(3.95)	3(3.9)	0(3.8)	1(3.7)	1(3.6)
**4**	0.01	0(0.11)	0(0.4)	0(0.59)	0(0.72)	0(0.77)	0(0.79)	0(0.78)	0(0.76)	0(0.74)	0(0.72)
**4**	0.001	0(0.011)	0(0.04)	0(0.059)	0(0.072)	0(0.077)	0(0.079)	0(0.078)	0(0.076)	0(0.074)	0(0.072)

**TOTAL**	0.05	206(187.9)	510(470.15)	684(597.4)	779(654.3)	784(679.8)	792(691.05)	744(697.2)	755(701)	802(703.25)	778(704.65)
**TOTAL**	0.01	49(37.58)	140(94.03)	186(119.48)	220(130.86)	225(135.96)	214(138.21)	230(139.44)	240(140.2)	258(140.65)	226(140.93)
**TOTAL**	0.001	22(3.758)	44(9.403)	71(11.948)	67(13.086)	78(13.596)	101(13.821)	90(13.944)	83(14.02)	83(14.065)	82(14.093)

The total number of significant tests obtained from the sliding window analysis for even numbered window sizes are presented with the expected numbers assuming a null distribution in parentheses.

**Table 3:** Number of overlapping significant windows between the analysis of all species and within *D. simulans*.

Chr	Pval	2	4	6	8	10	12	14	16	18	20
**X**	0.05	2(28,34)	4(64,73)	5(90,107)	2(101,128)	6(98,126)	5(109,122)	1(105,109)	5(101,117)	8(102,128)	3(110,121)
**X**	0.01	1(10,10)	2(15,19)	1(11,30)	0(15,32)	0(20,34)	0(26,34)	0(25,38)	0(26,37)	0(21,35)	0(25,31)
**X**	0.001	0(4,4)	0(7,6)	0(2,6)	0(4,9)	0(2,16)	0(7,11)	0(4,1)	0(2,7)	0(3,7)	0(2,7)
**2L**	0.05	2(31,25)	6(72,86)	10(88,129)	12(107,144)	19(117,155)	17(123,143)	18(130,154)	15(121,160)	20(126,171)	23(128,163)
**2L**	0.01	0(7,6)	2(15,25)	1(15,30)	0(16,33)	0(21,38)	1(25,36)	0(29,36)	2(23,42)	1(23,49)	1(27,41)
**2L**	0.001	0(1,2)	2(6,8)	1(3,10)	0(3,3)	0(3,5)	0(1,11)	0(6,14)	0(6,10)	0(7,14)	0(6,17)
**2R**	0.05	5(39,30)	3(104,86)	2(139,121)	6(150,146)	1(130,163)	1(123,171)	2(115,137)	7(122,130)	7(133,132)	5(159,120)
**2R**	0.01	0(5,7)	0(19,16)	0(30,35)	0(39,37)	0(42,41)	0(45,38)	0(49,27)	0(57,41)	0(58,43)	0(56,33)
**2R**	0.001	0(1,1)	0(5,2)	0(10,12)	0(18,14)	0(10,15)	0(21,12)	0(17,6)	0(37,9)	0(29,6)	0(35,11)
**3L**	0.05	6(36,37)	9(89,90)	9(114,107)	10(114,118)	14(123,127)	13(118,122)	22(132,129)	30(125,133)	26(119,134)	23(117,146)
**3L**	0.01	5(12,9)	4(16,20)	5(31,24)	6(25,35)	7(28,33)	10(26,35)	8(29,44)	12(21,43)	10(22,46)	1(11,44)
**3L**	0.001	3(7,5)	3(5,11)	3(8,9)	3(9,11)	5(8,12)	4(6,18)	2(5,25)	4(4,22)	2(3,22)	0(0,21)
**3R**	0.05	0(61,42)	4(113,96)	7(133,129)	13(143,149)	13(172,133)	20(172,139)	18(145,145)	16(149,137)	17(146,159)	20(158,155)
**3R**	0.01	0(13,8)	0(25,16)	0(30,24)	0(31,25)	1(36,27)	0(28,28)	3(29,35)	3(30,36)	0(27,36)	0(24,31)
**3R**	0.001	0(4,2)	0(7,3)	0(9,5)	0(10,4)	0(8,2)	0(8,8)	0(4,11)	0(10,11)	0(1,6)	0(5,9)
**4**	0.05	0(1,0)	0(2,0)	0(4,0)	0(7,0)	0(8,3)	0(9,3)	0(9,3)	0(5,0)	0(2,1)	0(1,1)
**4**	0.01	0(0,0)	0(1,0)	0(3,0)	0(5,0)	0(6,0)	0(5,0)	0(3,0)	0(1,0)	0(0,0)	0(0,0)
**4**	0.001	0(0,0)	0(1,0)	0(0,0)	0(0,0)	0(6,0)	0(5,0)	0(0,0)	0(0,0)	0(0,0)	0(0,0)

**TOTAL**	0.05	15(196,168)	26(444,431)	33(568,593)	43(622,685)	53(648,707)	56(654,700)	61(636,677)	73(623,677)	78(628,725)	74(673,706)
**TOTAL**	0.01	6(47,40)	8(91,96)	7(120,143)	6(131,162)	8(153,173)	11(155,171)	11(164,180)	17(158,199)	11(151,209)	2(143,180)
**TOTAL**	0.001	3(17,14)	5(31,30)	4(32,42)	3(44,41)	5(37,50)	4(48,60)	2(36,57)	4(59,59)	2(43,55)	0(48,65)

The first number in parentheses is the number of overlapping significant windows when comparing repeated analysis of the mean expression data for all species and the second number in parentheses is the number of overlapping windows when comparing repeated analysis of the *D. simulans *data.

While the mechanisms underlying the existence of clusters of co-evolution of expression among species and co-expression clusters within species cannot be resolved from these data, the existence of paralogous genes in close proximity can be ruled out as the major factor for the observed pattern. Paralogous genes in close proximity may be expected to produce the evolving clusters or within species co-expression clusters as a result of shared regulatory elements and/or maintained common functions. However, paralogs may also produce the pattern by cross-hybridizing to common probes on the microarray. If the second of these possibilities can explain a considerable proportion of clusters, this could mean the observed pattern was an artifact of the microarray assay. However, we find that very few paralogous gene sets are within either evolving clusters or co-expression clusters (between 1.4%–12.0% of evolving clusters identified using window sizes 2–20 and a p-value cutoff of 0.001 contain paralogous genes and 2.1%–8.5% of co-expression clusters within *D. simulans *contain paralogous genes; see Additional file 9). The majority of evolving clusters and co-expression clusters cannot therefore be explained by paralogous genes.

### Spatial, Adaptive, and Functional Distribution of Co-expression Clusters

To determine if co-expression or co-evolution of expression is related to the physical organization of genes within clusters, we investigated the spatial distribution of the clusters across the genome of *D. melanogaster*. While order of genes in clusters where expression is co-evolving are conserved across species in our analysis (see above), the physical location of these clusters relative to *D. melanogaster *need not be. Fortunately, the local spatial organization of genes is highly conserved across these species, which allows us to again use the heavily annotated *D. melanogaster *genome as a reference for our spatial analysis.

There are fewer co-evolving expression clusters in centromere proximal regions (heterochromatic centrometric regions were not included in our analysis). This was also observed for the within species co-expression clusters. Two hypotheses may explain this pattern. First, gene density tends to decline in regions proximal to the centromere [38], which may reduce the total number of gene clusters observed in these relatively gene depauperate regions. Second, centromere proximal regions have higher amounts of heterochromatin, which can dramatically affect gene expression [39,40]. Most euchromatic gene expression is suppressed in heterochromatin and natively heterochromatic genes are typically only expressed when surrounded by heterochromatin. Therefore, local shifts over evolutionary time in the heterochromatin content – which are common in centromere proximal regions – may inhibit the formation of clusters near centromeres [39].

Most genes in the genome are physically grouped together on the chromosome as determined by the coefficient of deviation (Table 4). In contrast, clusters of genes where there is co-expression or where there is co-evolution of expression tend to be more dispersed, which suggests that co-expression is not simply a function of gene density. Nor is it the result of local recombination rate; there is no relationship between the rate of recombination in *D. melanogaster *and the density of clusters (analysis not shown). This conflicts with the hypothesis that lower recombination tends to evolve among co-expressed genes [24,41]. This result may however be confounded by variation in recombination rate across the species analyzed.

**Table 4:** Comparisons of the spatial distributions of co-expression clusters.

	X	2L	2R	3L	3R
**Within Species**	1.16	1.47	1.24	0.96	1.1
**Between Species**	1.4	0.83	1.13	1.16	0.8
**Genome**	2.63	2.23	2.76	4.88	2.64

See Methods for full description. Typically, values around 1 suggest a random spatial distribution; values above 1 indicate clustering. Due to our masking approach, this was limited to genes included in our analysis. Interleaved and nested genes were ignored. "Within species" refers to the co-expression clusters found within *D. simulans*. "Between species" refers to the clusters found to be evolving among species. "Genome" refers to the genome wide estimate from the *D. melanogaster *genome.

In contrast to these large scale patterns, genes within evolving clusters do not show unusual *local *structural organization relative to the rest of the genome. For example, the genes in clusters where there is co-evolution of expression are not physically closer to each other relative to the rest of the genome (whole genome median spacing: 827 bases, whole genome mean spacing 4853 bases; cluster gene median spacing: 3573 bases; cluster gene mean spacing: 8503 bases). Likewise, there is no strand bias – genes are equally likely to be on either strand of DNA (+ strand: 118; - strand: 114). When considering pairs of genes – "window 2" clusters – there was no significant difference in the strand orientations of these pairs. Pairs of genes were essentially equally likely to both be on the same strand or on opposite strands in either the + - or -+ orientation (χ2 = 3.468, d.f. = 3, p-value = 0.3249). Nor were there significant runs of genes on the same strand among genes within the largest clusters (p-value = 0.45). There was similarly no unusual structural organization for clusters of co-expression within *D. simulans *compared to the rest of the genome.

However, as noted above, clusters where there is coordinated evolution of expression *among *species seldom correspond to clusters where there is co-expression *within *species. This fact suggests that the evolutionary and genetic forces affecting coordinated expression within and between species are distinct. If the evolution of co-expression clusters reflected a neutral process, we would expect the patterns of co-expression within species to be reflective of the patterns of co-expression between species. Instead, we see very different patterns within and between species. Between species, our analysis identifies groups of genes whose expression is correlated across evolutionary time. Natural selection, directional or purifying, could drive or preserve patterns of co-expression among genes. Directional selection, however, is the more likely explanation for the *diversification *in expression we observe across species. We tested this idea using data from Begun et al. 2007 [42]. Using polymorphism data in *D. simulan*s in conjunction with divergence data from *D. melanogaster *and *D. yakuba*, Begun et al. 2007 identified genes evincing recurrent directional selection using a McDonald-Krietman test [43]. These genes had normal levels of within species polymorphism, but high levels of between species divergence. We compared the frequency of genes with significant McDonald-Krietman tests (MKtest) within the clusters where there is co-evolution of expression to the whole genome empirical distribution (nominal threshold of p-value < 0.05). Genes within clusters have 27% more adaptively evolving genes than the genome average using a polarized MKtest, which does not confound evolution on two branches (p-value < 0.001; unpolarized test difference is only 4%). This result suggests that recurrent directional selection may be the evolutionary force shaping the evolution of co-evolving expression of neighboring genes. Recent work looking at a subset of the species we analyzed also suggests a tie between adaptive evolution of coding sequences and changes in gene expression [44]. The phenomena observed here may reflect that larger evolutionary process.

Our within species analysis of *D. simulans *identified clusters where expression is coordinated and we applied the MKtest analysis to these blocks of genes. In contrast to the among species analysis, the within *D. simulans *clusters lack genes evidencing recurrent adaptive amino acid evolution (nominal MKtest p-value < 0.05; polarized, 15% fewer adaptively evolving genes, p-value < 0.001; unpolarized 30% fewer adaptively evolving genes, p-value < 0.001). This paucity of significant MKtests may indicate a role for balancing selection in maintaining some, but not all, of these polymorphic within-species clusters. Regardless, distinctly different evolutionary forces appear to be operating to produce co-evolution of expression in clusters compared to co-expression clusters within species.

The seven species that we analyze are morphologically almost indistinguishable except for differences in male genitalia and in the case of *D. santomea *which has distinct pigmentation [26]. The co-evolving expression clusters are therefore not likely to be related to tissue specific expression of clustered genes underlying morphological divergence. We do, however, find there are more statistically over-represented GO categories involved in reproduction in clusters where there is co-evolution of expression and more genes involved in immune response for clusters evincing co-expression within species clusters (Table 5 and Additional file 9). Recurrent selection has repeatedly been shown to drive evolution of reproduction related genes, especially in males [45-47]. Thus it makes sense that our co-evolving expression clusters are enriched for both adaptively evolving and reproduction related genes. Similarly, a subset of immune response genes have high levels of nucleotide polymorphism within species [48-50], which is also consistent with our MKtest analysis of within species clusters.

**Table 5:** Over-representation of gene classes in clusters.

Among Species	*D. simulans*	Both
Male specific sperm protein	Protein of unknown function UPF0131	Glycoside hydrolase, family 22, lysozyme
Fruit fly testis-specific protein	Chorion 2	Lysozyme c
Developmental protein	Insect vitellogenin	LYZ1
Establishment of localization	Uncharacterized conserved protein	Antimicrobial
Exocrine system development	Antibacterial humoral response	Bacteriolytic enzyme
Glycerol kinase activity	Antimicrobial humoral response	Chorion
Localization	Cell wall catabolism	External encapsulating structure
Multigene family	Defense response to bacteria	Lysozyme activity
Reproduction	Eggshell formation	Polysaccharide degradation
Salivary gland determination	Female gamete generation	Sexual reproduction
Salivary gland development	Gametogenesis	Structural constituent of chorion (sensu Insecta)
Spermatogenesis	Glycosidase	
Tandem repeat	Humoral defense mechanism (sensu Protostomia)	
Transport	Humoral immune response	
Transporter activity	Hydrolase activity, acting on glycosyl bonds	
	Insect chorion formation	
	Membrane lipid metabolism	
	Phospholipid metabolism	
	Response to bacteria	
	Response to pest, pathogen or parasite	
	Signal	
	Structural molecule activity	
	Sulfation	
	Vitellogenesis	

Classes presented are those with a p-value < 0.05 as determined by DAVID (see Methods) for the clusters identified with a window size of 2 and a p-value cutoff of p < 0.001.

## Conclusion

To understand how complex phenotypes evolve, we need to understand how interacting genes evolve. We have analyzed expression microarray data from species in the *Drosophila melanogaster *subgroup, including data from ten distinct *D. simulans *lines and identified blocks of genes where there is coordinated evolution of expression among species. We have also identified clusters where there is co-expression within the species *D. simulans*. Our work shows that coordinated evolution of expression among physically adjacent genes among species and co-expression among adjacent genes within species is common. In general, there is little correspondence between clusters where there is co-evolution of expression and those clusters where there is co-expression within species for the species analyzed. The evolutionary forces shaping these two types of clusters are clearly different. Our analysis of co-expression within a species showed no relationship between adaptive evolution at the sequence level and expression of genes within clusters. In contrast, adaptive sequence evolution is associated with those genes in clusters where expression is evolving in a coordinated manner. In sum, we find that just as expression of genes is not independent of physical location of genes within a species, the evolution of expression of many genes is not independent of the evolution of their neighbors. We also show that this result is not simply the byproduct of clusters of duplicated genes. Our analysis suggests that clusters of genes where there is co-evolution of expression may be natural units for quantifying and understanding the evolution of transcriptomes.

## Methods

### Data Collection

For all species, flies were raised under the standardized conditions as described in Nuzhdin et al. 2004 [25]. Under these conditions, a component of the variation in gene expression will reflect evolved differences among species. For males three days post-pupation, RNA was extracted from whole adult tissue using 20 individual flies per replicate. Assays of transcript abundance were carried out using *Drosophila *1.0 Affymetrix GeneChip Arrays using the recommended protocols (Affymetrix Inc, Santa Clara, CA). Assays were carried out on three independent (biological) replicates per species for a single line of the following five additional species: *D. yakuba *(Tuscon Stock Center Number: 14021-0261.00), *D. santomea *(TSCN: 14021-0271.00), *D. teissieri *(TSCN: 14021-0257.00), *D. mauritiana *(David 105, TSCN: 14021-0241.01), *D. sechellia *(Roberstson, TSCN: 14021-0248.21). These data were combined with data previously collected for *D. melanogaster *and *D. simulans *under the same conditions that used the same Affymetrix GeneChip Array [25]. Analyses of the entire data set therefore included a total of 48 independent transcript assays covering seven *Drosophila *species in the *D. melanogaster *subgroup (Figure 1). Array data have been deposited in GEO repository (Series record GSE7873). Processed data after background correction and normalization without masking are available in the Additional files (Additional file 2).

Note that the samples assayed for *D. melanogaster *reflect an even genotypic contribution of 10 isogenic lines developed from a wild population (Winters, CA) and crossed in a round-robin design. Variation in transcript abundance in *D. melanogaster *therefore includes variation due to polymorphism and measurement error. For *D. simulans*, three replicate arrays were used to assay each of 10 round-robin crosses between 10 isogenic lines developed from the same population. Each group of three replicates for *D. simulans *can therefore be used as an estimate of the expression levels associated with individual (heterozygous) genotypes and the variation assayed can be attributed to measurement error. Variation in transcript abundance among these *D. simulans *genotypes provides an estimate of the within species genetic variation in gene expression. For all other species, variation among replicate arrays includes only measurement error. Analysis of the means of transcript abundance among these seven different species therefore provides an estimate of the variation in gene expression that has evolved among species.

### Microarray Probe Masking and Normalization

The *Drosophila *1.0 Affymetrix GeneChip is designed with probe sets for 14,010 locations of the *D. melanogater *genome with sets usually containing 14 pairs of 25-nucleotide probes. For each pair, one of the pair is a 'perfect' match (PM) to the *D. melanogaster *reference sequence and the other differs only at the 13th base and is the mismatch probe (MM), the latter used to account for non-specific hybridization. We expect many of the PM probes to not be perfect matches for other species. To account for these effects, we removed all probe pairs where the PM probe is not an exact sequence match to *D. yakuba*. *D. melanogaster *and *D. yakuba *shared a common ancestor ~6 million years ago [26] and reflect the broadest taxonomic sampling of the species analyzed. Probe homology between them makes the probes likely to be perfect matches for all species analyzed. Indeed comparison of *D. yakuba *to *D. simulans *shows that this is true for 87.4% of genes included in our analysis. For maximum power, we would mask only those probes that were divergent in a particular species, however, for several practical reasons we did not do this: 1. we found that including the *D. simulans *mismatches had little effect, once the *D. yakuba *was masked, 2. adding *D. sechellia *has little effect vs *D. simulans*, 3. as genome sequences are not available for all species in our study, we cannot make masks for all taxa. From an analysis point of view, using multiple masks is problematic as the power and precision of expression estimation would then vary across taxa. We therefore opted for a conservative approach of masking all taxa based on the most divergent genome, which should catch the most egregious cases. This approach is conservative as unmasked divergent probes typically, but not always, lead to over-estimates of expression (see below). As explained below, our analysis looks for genes showing consistent patterns of expression across taxa among expressed genes (e.g. those that appear to be above background). Including the occasional mismatch likely reduces our power to detect such a pattern, not create it. Thus, positive results in our analysis are probably robust to the spurious noise caused by mismatched probes.

To identify probes for removal (masking) we used BLAST to compare the Affymetrix target sequences from this array to identify homologous regions in release 2.0 of the *D. yakuba *genome. We conducted this analysis on each chromosome arm to minimize spurious matches. Contigs with the best matches to the target sequences were then compared to the Affymetrix probe sequences. Full matches were then extracted and the percent match calculated and the locations and types of mismatches noted. After probe removal, a total of 7024 probe sets were still represented on the array, with the following breakdown for number of probes removed (0–3) 85, (4–6) 396, (7–9) 1701, (10–12) 4842. Note that if (13) or (14) probes were removed, the probe set was not included in the analysis.

A number of previous analyses have found that highly expressed genes tend to evolve at a slower rate [51-53]. This will result in two effects. First, we expect genes with low expression to have more probes removed and on average will therefore have higher standard errors. This will tend to reduce our chances of identifying co-expression clusters for these genes. Second, a larger proportion of genes with low expression will be removed from the analysis completely since none of the probes will be homologous between *D. melanogaster *and *D. yakuba*. If higher expressed gene are comparatively enriched for co-expression clusters within species, our analysis will be considering a subset of genes that are potentially enriched for within species clusters. Given that our permutation approach permutes among genes for which we have data this may lead to inflation of the number of clusters identified as significant at different cutoffs. However, testing the null hypothesis of the existence of co-expression clusters or clusters where there is co-evolution of expression is still valid in this case and the relative ordering of significance will not be affected. We therefore expect the clusters identified as the most significant in our analysis to reflect the most likely candidates for being true co-expression clusters or clusters where there is co-evolution of expression.

Background correction and normalization of our masked set was carried out using packages available in Bioconductor [54]. We found that a Loess smoothing followed by "mas5" and "liwong" [in Bioconductor: liwong or mas5(normalize(, method="loess"))] produced the best correction for intensity dependent trends as determined by MA plots (see Additional file 1). Results of our co-expression analysis for these two normalizations did not differ qualitatively and in the following we only present the results for MAS5. Note that the Loess smoothing has a stochastic component which results in slightly different measures for a given run. We repeated our Loess + normalization five times to assess the effects of this stochastic component. Differences when running the co-expression analysis on these were found to be minimal and we randomly chose one outcome for further analysis.

**Figure 4:**
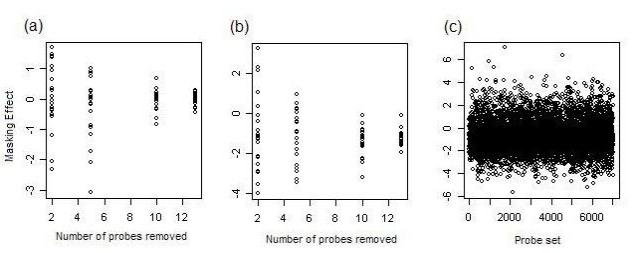
Effects of probe masking on the estimated expression level. Each circle reflects the slope of the regression divided by mean squared error for a single probe set for a regression using all 48 samples: A) Effect of masking random probes for probe sets where all 14 probes are perfect matches for both *D. melanogaster *and *D. yakuba*, B) Effect of masking in probe sets where probes masked have diverged between *D. melanogaster *and *D. yakuba *(all other probes in a set are perfect matches for both species), C) Effect of masking for all probe sets included in the analysis.

Obtaining accurate transcript abundance measures using the masking approach depends on the assumptions that using a subset of the 14 probe pairs does not dramatically bias the final measure of transcript abundance. Conversely, using a known PM probe that is diverged among species is expected to produce a biased measure. To assess the first of these assumptions, we compared the level of expression for in *D. yakuba *for 21 genes where all 14 probesets in *D. yakuba *were also PM in *D. melanogaster*. We analyzed the effect of removing 1, 5, 9, and 12 probes on the final expression measure using Loess + MAS5 (Figure 4A). We found that while the effect of probe removal can have a significant effect on expression (mean *R*^2 ^for removal of 1, 5, 9, and 12 probes were 0.008, 0.0236, 0.1449, 0.1718, respectively), the effect is equally likely to be an increase or decrease – that is, the direction of the change in effect tends to be random (p-value >> 0.05 for sign tests). For the second assumption, we compared the gene expression levels after masking sets of 21 randomly chosen genes that had 1, 5, 9, and 12 mismatched probes when compared to *D. yakuba *to the results when not masking these probes (Figure 4B). The results were even more significant (mean *R*^2 ^for removal probes were 0.2887, 0.3480, 0.3333, 0.4310, respectively) but biased towards producing smaller values when masking (sign test p-value << 0.01). In other words, including mismatched probes tends to bias estimates of expression upward. This is the overall trend when comparing the masked to unmasked measures for the probe sets included in the analyses described below (Figure 4C). We suspect this result occurs because a small subset of probes hybridized heterologous cRNA better than homologous cRNA. This subset of probes has a disproportionate effect on the mean because of the inability of the technology to detect differences between poorly hybridizing probes and very poorly hybridizing probes.

We used Ensembl (CG/CR) annotation provided by Affymetrix and merged these with the *Drosophila melanogater *genome annotation (release 4.3) retaining genes with protein coding regions (CDS, protein, and gene). We used the *D. melanogaster *annotation to determine gene order. In total, our gene ordering included 14517 genes. After masking, we obtained transcript abundances for 7024 that were dispersed relatively evenly across the entire genome and generally tracked gene density: X: 1118, 2L: 1236, 2R: 1421, 3L: 1408, 3R: 1811, 4: 30. Our co-expression analysis was carried out on the entire ordering of 14517 genes handling missing data as described below. The genes along each chromosome were ordered based on the start coordinates of *D. melanogaster *and were numbered consecutively (See Additional file 3).

We performed ANOVAs to assess whether there was significant variation among the 7024 genes among species and within *D. simulans*. For a significance level of 0.05 we found 5344 (among) and 6825 (within) tests and at 0.001 we found 3111 and 2887, respectively. Thus, there appears to be detectably significant variation for most of the genes consider in our analysis.

### Evolutionary Co-Expression Analysis

Our approach for identifying clusters where there is co-evolution of expression is closest to the analysis of correlation approach used by Spellman and Rubin 2002 with the following two differences: 1. use of an eigenvalue ratio statistic to summarize correlations within a window instead of the mean correlation, and 2. analysis of all window sizes from 2–20 instead of limiting analysis to a single window size of 10 genes. For the co-expression analysis, we calculated the mean value of each gene for a given species using the *total *set of the arrays used to assay that species. We then carried out a sliding window analysis of the correlations among pairs of consecutive genes, i.e. correlations for pairs of genes were considered where there were seven 'species' observations per gene. Our test statistic for each window is calculated as follows: 1. perform a principal components analysis (i.e. calculate eigenvectors) on the correlation matrix for all the genes in the window, 2. calculate the ratio of the first eigenvalue compared to the sum of all of the eigenvalues. Intuitively, this statistic reflects the fraction of variation along the first principal component. As an example of the structure of correlation captured by this statistic, consider the following correlation matrices:

$$\begin{array}{ccc} \left[\begin{array}{cccc} 1.0 & 0.8 & 0.0 & 0.0\\ 0.8 & 1.0 & 0.0 & 0.0\\ 0.0 & 0.0 & 1.0 & 0.8\\ 0.0 & 0.0 & 0.8 & 1.0 \end{array}\right] & \left[\begin{array}{cccc} 1.0 & 0.8 & 0.8 & 0.7\\ 0.8 & 1.0 & 0.7 & 0.8\\ 0.8 & 0.7 & 1.0 & 0.8\\ 0.7 & 0.8 & 0.8 & 1.0 \end{array}\right] & \left[\begin{array}{cccc} 1.0 & 0.8 & -0.8 & -0.7\\ 0.8 & 1.0 & -0.7 & -0.8\\ -0.8 & -0.7 & 1.0 & 0.8\\ -0.7 & -0.8 & 0.8 & 1.0 \end{array}\right] \end{array}$$

The value of our statistic for these matrices is 0.45, 0.825, and 0.825 respectively. Note that this statistic tends to reflect our intuition about the extent of correlation among the (in this case) four genes in the window. In the first case, gene pairs {1,2} and {3,4} are highly correlated but there is no correlation between genes in different pairs. This is therefore a case where the entire set of genes are not highly correlated compared to the case of the second matrix where every gene is highly correlated with every other gene in the window, and the statistic reflects this structure (0.825 > 0.45). Also, note that it does not matter if the correlations are negative or positive, only that the genes are acting as a *correlated *group, i.e. the second and third matrices have the same value of the statistic.

The co-expression analysis proceeded by calculating the eigenvalue ratio statistic for each consecutive (overlapping) window along each individual chromosome for window sizes of 2–20. Note that in most cases, missing data caused the total number of genes in a given window to be less than the window size. A direct consequence of this is that larger window sizes often included the same number of gene sets as smaller window sizes for specific windows. Each window size in the analysis should therefore not be considered as analyzing the exact number of genes of the window size but as a relative measure considering more genes on average as the window size increases.

To identify specific sets of genes that have more extreme co-expression for a given window size, we randomly permuted data within each chromosome for genes where we had data. We did this randomization 1000 times and repeated the sliding window analysis each time. We then counted the number of consecutive windows that had values of the statistic greater than the top 5%, 1%, and 0.1% of these random sets. In many cases, these groups of genes at the various cutoffs overlap. We compared the number of significant tests at these cutoffs to the expected number we would obtain if there were no true significant tests. This is the expected number of false discoveries and the ratio of these two is an estimate of the false discovery rate [34]. We present the number of significant windows (Table 1), the p-values associated with each window (Additional file 4), and merged significant windows at the 0.05 significance level (Additional file 5).

We additionally used a randomization approach to assess whether the genome-wide number of clusters where expression is co-evolving is greater than we would expect at random. We repeated the analysis and 1000 permutations for 100 random data sets, where again, we randomly re-ordered the genes within a chromosome for which we had data (i.e. we kept the missing data structure intact). We then repeated the co-expression analysis for window sizes 2, 5, 10, and 20 and counted the number of windows that had p-values less than 0.001 of the 1000 random sets of a given window size as described in the previous paragraph, i.e. our statistic used to assess significance is the number of windows identified at a given cutoff in our original data (Table 1). We used the 0.001 cutoff since this corresponds to the lowest FDR levels associated with the number of significant windows that we identified and we therefore have the greatest confidence that many of these reflect true clusters where expression is co-evolving. We did not perform this analysis for all window sizes given computational constraints.

A potential concern with this approach is that a single species with dramatically different gene expression levels may be driving the pattern across all species, i.e. not all species are evolving so co-expression is species specific. To assess this possibility we performed the sliding window analysis after removing a single species and compared the results to the analysis including all species. A dramatic difference between the number of regions identified would indicate that the overall pattern may be lineage/species specific. We determined the number of windows that were below a given cutoff in both the analysis of all species and for the analysis with one species removed. We present these results in Additional file 6. As a metric for comparison, we also determined the number of windows below cutoffs for the original analysis of all species and for a repeated analysis of all species (i.e. an additional independent 1000 permutations). Results are presented in Table 3.

An additional concern with our analysis is we are using the gene ordering of *D. melanogaster*. For species in the *D. yakuba *branch there are known to be a considerable number of rearrangements affecting gene order. For such locations, our analysis is not being performed on genes that are physically adjacent for all species. If our analysis is identifying adjacent genes that are more correlated than other gene sets, we would not expect to find highly correlated genes that are interrupted by these breakpoints more often than expected by chance. To assess this possibility, we mapped *D. yakuba *breakpoints to positions of the regions where expression is co-evolving. We generated this file by computationally comparing the locations of all *D. yakuba *genes in our array data set to the position of their *D. melanogaster *homolog. Anywhere that a block of *D. yakuba *genes shifted to a new position was recorded and marked as an inversion breakpoint. These breakpoints are essentially the same as those noted in Lemeunier and Ashburner 1976 [55]. Like Ranz *et al.* 2007, we find no effect of the presences of breakpoints on the presence or absence of cluster.

### Within Species Co-Expression Analysis

The entire co-expression analysis was also repeated to identify co-expression domains within *D. simulans*. We used the *D. yakuba *mask file for this analysis so that the results are directly comparable to the results among species and conservative as we likely mask more genes than necessary. These data consisted of three replicate array assays for 10 crosses between *D. simulans *lines [56]. While expression array analysis of each cross therefore does not assess the transcript abundance variation associated with a single genotype, assay of these crosses provides a reasonable estimate of the variation observed in this *D. simulans *population. We performed the co-expression analysis on the mean value associated with each of the genotype crosses. We present the number of significant windows (Table 2), the p-values associated with each window (Additional file 7), and merged significant windows at the 0.05 significance level (Additional file 8). We compared these regions to those found among species by comparing the number of windows with p-values below 0.05, 0.01, and 0.001. As a metric for comparison, we also determined the number of windows below cutoffs for a repeated analysis of *D. simulans *(Table 3).

The evolutionary analysis of transcript abundance considers a single representative line per species with the exception of *D. simulans*. Thus, the observed variation between species – in principle – may only reflect the typical *intra*-specific variation between any two lines within species. To assess this possibility, we performed individual ANOVAs for each gene comparing the values of the *D. simulans *crossed-genotypes to the other six species considered as a group. We compared the distribution of p-values for this analysis to ANOVAs for the same partition where each array was assigned to a partition at random. We identified far more p-values at a given cutoff for the *D. simulans *vs. other species indicating that the variation we are analyzing between species is far greater than the variation we observed within the 10 combined-genotypes of *D. simulans *(6825 significant tests as compared to 5291 in the random assignment at 0.05, 2887 significant compared to 2022 random at 0.001).

We compared the relative locations of clusters we identified to be evolving across species and within *D. simulans *at the 0.05 and 0.001 cutoffs to the intra-species clusters identified in *D. melanogaster *by Spellman and Rubin 2002 [12]. Since Spellman and Rubin 2002 reported a set of clusters representing merged overlapping windows using a window size of 10 genes, we calculated merged windows for a window size of 10 for our among and within species analysis and compared the overlap between these windows. Note that in the Spellman and Rubin 2002 study, the co-expression among genes expressed at different developmental stages and under different environmental conditions was analyzed while in the current study, only expression at the adult stage was considered. Not surprisingly, we found little correspondence between the co-expression clusters identified within *D. simulans *when compared to the results of Spellman and Rubin 2002.

### Additional Representation Analyses

We considered whether the existence of paralogous genes could explain the existence of clusters. Tandem pairs of duplicated genes were identified by sequentially estimating the degree of similarity of all neighboring pairs of genes on all major chromosomes using the gene ordering of FLYBASE v5 (i.e. we are using *D. melanogaster *as a reference). At a local scale these syntenic relationships should be preserved across taxa [33]. The first transcript of each gene (e.g. PA) was translated and pairs of proteins were then aligned with bl2seq, a version of BLAST that is optimized for pair wise alignments. Genes with 85% amino acid similarity were considered tandem duplicates. Typically, more than 90% of these genes were greater than 90% similar in amino acid sequence. We then determined the number of cases where paralogs gene pairs were located within clusters identified among species and within *D. simulans *(Additional file 9).

We also considered whether clusters either within *D. simulans *or among species tend to be located at regions of high gene density. We calculated the gene density in a co-evolving or co-expression window from the start of the first gene to the start of the last gene in that cluster. We then calculated the empirical distribution of like sized windows across that chromosome arm. The mean gene density of clusters was then compared to this distribution.

We used the coefficient of dispersion (CD=s2X¯), a commonly used as a measure of spatial clustering, to look at large scale spatial patterns of both genes and clusters. For genes, the distance (in nucleotides) from the end of one gene to the start of the next was calculated for each locus. For clusters, we used the distance in nucleotides from the end of one cluster to the start of the next. The mean, variance, and *CD *were calculated for each chromosome arm for both genes and clusters. *CD *much greater than one normally suggests clumping; *CD *less than one indicates over dispersal.

We mined FLYBASE for genetic map data and physical map data. From these data we inferred regional recombination rates. We then compared the mean recombination rate of the genes in our clusters to the genome average. There was no significant difference.

We used the *D. melanogaster *annotation to see if pairs of genes within clusters tended to be on the same strand as each other. For simplicity, we limited this analysis to clusters of only two genes. If shared *cis*-regulatory regions were critical to coordinated expression evolution, we may expect that the genes would tend to be on the same stand.

We used the DAVID analysis tool [57] to determine which gene ontology classes were over-represented in identified clusters. We did this for both clusters identified across species and within *D. simulans *at window size of 2 and 10 using the 0.05 and 0.001 cutoffs.

### Evolutionary Analysis

Begun et al. 2007 identified genes in the *D. simulans *genome that deviated from the standard neutral model in a manner consistent with recurrent directional selection. They performed both polarized and unpolarized McDonald-Krietman tests [43]. Polarized tests only identify genes evolving "adaptively" along the *D. simulans *lineage. Unpolarized tests confound divergence across both *D. melanogaster *and *D. simulans *lineages, but may capture genes adaptively evolving in both species. Both data sets were used to see if genes within clusters – either within *D. simulans *or among taxa – are enriched for adaptively evolving genes. We employed a re-sampling approach to determine if one particular subset of genes was enriched (10,000 samples for each analysis). As only those genes for which sufficient polymorphism data were used Begun et al.'s analysis, only a subsample of the genome was tested (unpolarized *N *= 6704; polarized *N *= 2653). As detailed in Begun et al. 2007, if a correction for multiple testing, such as a Bonferroni correction, is used very few genes in the genome-wide comparison are significant. Thus, for the resampling analysis we set a nominal p-value threshold for the McDonald-Krietman tests; enrichment is thus defined as having a significantly greater number of McDonald-Krietman tests with p-values below this nominal threshold.

## Authors' contributions

JGM and SVN collected the data analyzed. CDJ performed the probe masking, the evolutionary analysis of adaptive evolution in co-expression clusters, tandem duplicate identification, and the spatial analysis of cluster distribution. JGM and FY assessed effects of normalization and masking on microarray data and performed the co-expression, permutations, and related analyses. JGM and CDJ wrote the paper.

## Supplementary Material

Additional File 1 MA plots. Representative MA plots comparing array results within *D. simulans *and across species. Click here for file

Additional File 2 Array data without mask. Processed data for the 48 Affymetrix arrays used to analyze the seven species after Loess smoothing and background correction + normalization using MAS5 without masking (see text). The list includes genes with protein coding regions from the *Drosophila melanogaster *genome annotation (release 4.3). Columns are as follows: A. Ensembl - the CG/CR annotation provided by Affymetrix, B. Chromosome, C. Strand - + sense/- anti-sense, D/E. Start/Stop from release 4.3, F. Affymetrix Probe Set ID, G-I. Replicates for *Drosophila melanogaster*, J-L. *D. sechellia*, M-0. *D. mauritiana*, P-R., *D. teissieri*, S-U. *D. yakuba*, V-Y. *D. santomea*, Z-BB Replicates for the 10 crosses (3 each) of *D. simulans*. Click here for file

Additional File 3 Array data with mask. Processed data for the 48 Affymetrix arrays used to analyze the seven species after Loess smoothing and background correction + normalization using MAS5 after probe masking (see text). A-F. Same as Additional file 2, G. Number of probes in the probe set removed by masking, H-N. Mean values for the seven species, O-BJ. see Additional file 2. Click here for file

Additional File 4 Among species sliding window analysis. p-values determined for each window in the among species analysis. The p-value is listed on the row of the first gene of the window considered. A-G. Same as Additional file 3. H. number of genes used to calculate values, I. eigenvalue statistic, J. p-value, for a window size of 2 genes, L-BL. same for window sizes 3–20. Click here for file

Additional File 5 Merged windows among species 1. Merged windows found to be significant at p-value = 0.05 in the across species analysis. A-G. Same as Additional file 3. H-Z. Window sizes 2–20. Click here for file

Additional File 6 Merged windows among species 2. Results of repeating the sliding window analysis when removing one species at a time. Each window size presents the number of significant windows found in the analysis of all species and the numbers in parentheses reflect the range of significant windows identified when repeating the analysis removing one species at a time. Click here for file

Additional File 7 *Drosophila simulans *sliding window analysis. p-values determined for each window in the *D. simulans *analysis. The p-value is listed on the row of the first gene of the window considered. A-BL same as Additional file 4. Click here for file

Additional File 8 Merged windows *Drosophila simulans *1. Merged windows found to be significant at p-value = 0.05 in the *D. simulans *analysis. A-G. Same as Additional file 5. H-Z. Window sizes 2–20. Click here for file

Additional File 9 Merged windows *Drosophila simulans *2. Sets of paralogous genes and representation of these sets among significant windows identified at p-value < 0.001. A-C. Chromosome location and names of paralogous gene pairs. Note that some of these combine into larger paralog gene sets (highlighted in yellow or green). D-H. Results for the among species analysis ("AS"), *i.e*. window size used in the analysis, number of significant windows (numbers correspond to Table 1), number of these windows that contain paralogous, the number of paralogous in significant windows, the number of paralogs not in significant windows. I-M. Analogous results for the analysis within *D. simulans *("WS"). Click here for file

Additional File 10 Over-represented gene classes. Classes of genes over-represented in co-expression clusters as identified using DAVID (see text). Results are presented for the analysis among species and for the analysis within *D. simulans *for window size of 2 at the p-value cutoff of p-value = 0.001. Classes with p-values < 0.05 as determined by DAVID are presented. Click here for file

## References

[B1] Hurst LD Pal C Lercher MJ The evolutionary dynamics of eukaryotic gene order Nature Reviews Genetics 2004 5 299 310 10.1038/nrg1319 15131653

[B2] Lawrence JG Shared strategies in gene organization among prokaryotes and eukaryotes Cell 2002 110 407 413 10.1016/S0092-8674(02)00900-5 12202031

[B3] Oliver B Misteli T A non-random walk through the genome Genome Biol 2005 6 4 214 10.1186/gb-2005-6-4-214 15833129 PMC1088951

[B4] Cohen BA Mitra RD Hughes JD Church GM A computational analysis of whole-genome expression data reveals chromosomal domains of gene expression Nature Genetics 2000 26 183 186 10.1038/79896 11017073

[B5] Hurst LD Williams EJB Pal C Natural selection promotes the conservation of linkage of co-expressed genes Trends in Genetics 2002 18 604 606 10.1016/S0168-9525(02)02813-5 12446137

[B6] Williams EJB Bowles DJ Coexpression of neighboring genes in the genome of arabidopsis thaliana Genome Research 2004 14 1060 1067 10.1101/gr.2131104 15173112 PMC419784

[B7] Denver DR Morris K Streelman JT Kim SK Lynch M Thomas WK The transcriptional consequences of mutation and natural selection in caenorhabditis elegans Nat Genet 2005 37 544 548 10.1038/ng1554 15852004

[B8] Lercher MJ Blumenthal T Hurst LD Coexpression of neighboring genes in caenorhabditis elegans is mostly due to operons and duplicate genes Genome Research 2003 13 238 243 10.1101/gr.553803 12566401 PMC420373

[B9] Roy PJ Stuart JM Lund J Kim SK Chromosomal clustering of muscle-expressed genes in caenorhabditis elegans Nature 2002 418 975 979 12214599 10.1038/nature01012

[B10] Blumenthal T Evans D Link CD Guffanti A Lawson D Thierry-Mieg J Thierry-Mieg D Chiu WL Duke K Kiraly M Kim SK A global analysis of caenorhabditis elegans operons Nature 2002 417 851 854 10.1038/nature00831 12075352

[B11] Stolc V Gauhar Z Mason C Halasz G van Batenburg MF Rifkin SA Hua S Herreman T Tongprasit W Barbano PE Bussemaker HJ White KP A gene expression map for the euchromatic genome of drosophila melanogaster Science 2004 306 655 660 10.1126/science.1101312 15499012

[B12] Spellman PT Rubin GM Evidence for large domains of similarly expressed genes in the drosophila genome J Biol 2002 1 5 10.1186/1475-4924-1-5 12144710 PMC117248

[B13] Belyakin SN Christophides GK Alekseyenko AA Kriventseva EV Belyaeva ES Nanayev RA Makunin IV Kafatos FC Zhimulev IF Genomic analysis of drosophila chromosome underreplication reveals a link between replication control and transcriptional territories Proc Natl Acad Sci USA 2005 102 8269 8274 10.1073/pnas.0502702102 15928082 PMC1149430

[B14] Reymond A Marigo V Yaylaoglu MB Leoni A Ucla C Scamuffa N Caccioppoli C Dermitzakis ET Lyle R Banfi S Eichele G Antonarakis SE Ballabio A Human chromosome 21 gene expression atlas in the mouse Nature 2002 420 582 586 10.1038/nature01178 12466854

[B15] Mijalski T Harder A Halder T Kersten M Horsch M Strom TM Liebscher HV Lottspeich F de Angelis MH Beckers J Identification of coexpressed gene clusters in a comparative analysis of transcriptome and proteome in mouse tissues Proc Natl Acad Sci USA 2005 102 8621 8626 10.1073/pnas.0407672102 15939889 PMC1143582

[B16] Purmann A Toedling J Schueler M Carninci P Lehrach H Hayashizaki Y Huber W Sperling S Genomic organization of transcriptomes in mammals: Coregulation and cofunctionality Genomics 2007 89 580 587 10.1016/j.ygeno.2007.01.010 17369017

[B17] Caron H van Schaik B van der Mee M Baas F Riggins G van Sluis P Hermus MC van Asperen R Boon K Voute PA Heisterkamp S van Kampen A Versteeg R The human transcriptome map: Clustering of highly expressed genes in chromosomal domains Science 2001 291 1289 10.1126/science.1056794 11181992

[B18] Singer GA Lloyd AT Huminiecki LB Wolfe KH Clusters of co-expressed genes in mammalian genomes are conserved by natural selection Mol Biol Evol 2005 22 767 775 10.1093/molbev/msi062 15574806

[B19] Khaitovich P Muetzel B She XW Lachmann M Hellmann I Dietzsch J Steigele S Do HH Weiss G Enard W Heissig F Arendt T Nieselt-Struwe K Eichler EE Paabo S Regional patterns of gene expression in human and chimpanzee brains Genome Research 2004 14 1462 1473 10.1101/gr.2538704 15289471 PMC509255

[B20] Liu C Ghosh S Searls DB Saunders AM Cossman J Roses AD Clusters of adjacent and similarly expressed genes across normal human tissues complicate comparative transcriptomic discovery Omics-a Journal of Integrative Biology 2005 9 351 363 10.1089/omi.2005.9.351 16402893

[B21] Oliver B Parisi M Clark D Gene expression neighborhoods J Biol 2002 1 1 4 10.1186/1475-4924-1-4 12144705 PMC117247

[B22] Poyatos JF Hurst LD Is optimal gene order impossible? Trends in Genetics 2006 22 420 423 10.1016/j.tig.2006.06.003 16806566

[B23] Lercher MJ Chamary JV Hurst LD Genomic regionality in rates of evolution is not explained by clustering of genes of comparable expression profile Genome Research 2004 14 1002 1013 10.1101/gr.1597404 15173108 PMC419778

[B24] Semon M Duret L Evolutionary origin and maintenance of coexpressed gene clusters in mammals Mol Biol Evol 2006 23 1715 1723 10.1093/molbev/msl034 16757654

[B25] Nuzhdin SV Wayne ML Harmon KL McIntyre LM Common pattern of evolution of gene expression level and protein sequence in drosophila Molecular Biology and Evolution 2004 21 1308 1317 10.1093/molbev/msh128 15034135

[B26] Ashburner M Ball CA Blake JA Botstein D Butler H Cherry JM Davis AP Dolinski K Dwight SS Eppig JT Harris MA Hill DP Issel-Tarver L Kasarskis A Lewis S Matese JC Richardson JE Ringwald M Rubin GM Sherlock G Gene ontology: Tool for the unification of biology. the gene ontology consortium Nat Genet 2000 25 25 29 10.1038/75556 10802651 PMC3037419

[B27] Fukuoka Y Inaoka H Kohane IS Inter-species differences of co-expression of neighboring genes in eukaryotic genomes Bmc Genomics 2004 5 10.1186/1471-2164-5-4 PMC331401 14718066

[B28] Lercher MJ Hurst LD Co-expressed yeast genes cluster over a long range but are not regularly spaced Journal of Molecular Biology 2006 359 825 831 10.1016/j.jmb.2006.03.051 16631793

[B29] Turkheimer FE Roncaroli F Hennuy B Herens C Nguyen M Martin D Evrard A Bours V Boniver J Deprez M Chromosomal patterns of gene expression from microarray data: Methodology, validation and clinical relevance in gliomas BMC Bioinformatics 2006 7 526 10.1186/1471-2105-7-526 17140431 PMC1698583

[B30] Mezey JG Houle D The dimensionality of genetic variation for wing shape in drosophila melanogaster Evolution Int J Org Evolution 2005 59 1027 1038 16136802

[B31] Timm NH Applied Multivariate Analysis 2002 Springer-Verlag, New York

[B32] Mouse Genome Sequencing Consortium Initial sequencing and comparative analysis of the mouse genome Nature 2002 420 520 562 10.1038/nature01262 12466850

[B33] Ranz JM Maurin D Chan YS von Grotthuss M Hillier LW Roote J Ashburner M Bergman CM Principles of genome evolution in the drosophila melanogaster species group PLoS Biol 2007 5 e152 10.1371/journal.pbio.0050152 17550304 PMC1885836

[B34] Manly KF Nettleton D Hwang JTG Genomics, prior probability, and statistical tests of multiple hypotheses Genome Research 2004 14 997 1001 10.1101/gr.2156804 15173107

[B35] Storey JD Tibshirani R Statistical significance for genomewide studies Proc Natl Acad Sci USA 2003 100 9440 9445 10.1073/pnas.1530509100 12883005 PMC170937

[B36] Balazsi G Kay KA Barabasi AL Oltvai ZN Spurious spatial periodicity of co-expression in microarray data due to printing design Nucleic Acids Research 2003 31 4425 4433 10.1093/nar/gkg485 12888502 PMC169875

[B37] Kluger Y Yu HY Qian J Gerstein M Relationship between gene co-expression and probe localization on microarray slides Bmc Genomics 2003 4 49 10.1186/1471-2164-4-49 14667251 PMC317287

[B38] Noor MA Cunningham AL Larkin JC Consequences of recombination rate variation on quantitative trait locus mapping studies. simulations based on the drosophila melanogaster genome Genetics 2001 159 581 588 11606535 10.1093/genetics/159.2.581 PMC1461817

[B39] Yasuhara JC Wakimoto BT Oxymoron no more: The expanding world of heterochromatic genes Trends Genet 2006 22 330 338 10.1016/j.tig.2006.04.008 16690158

[B40] Weiler KS Wakimoto BT Heterochromatin and gene expression in drosophila Annu Rev Genet 1995 29 577 605 10.1146/annurev.ge.29.120195.003045 8825487

[B41] Clement Y Tavares R Marais GA Does lack of recombination enhance asymmetric evolution among duplicate genes? insights from the drosophila melanogaster genome Gene 2006 385 89 95 10.1016/j.gene.2006.05.032 17049187

[B42] Begun DJ Holloway AK Stevens K Hillier LW Poh YP Hahn MW Nista PM Jones CD Kern AD Dewey CN Pachter L Myers E Langley CH Population genomics: Whole-genome analysis of polymorphism and divergence in drosophila simulans PLoS Biol 2007 5 e310 10.1371/journal.pbio.0050310 17988176 PMC2062478

[B43] McDonald JH Kreitman M Adaptive protein evolution at the adh locus in drosophila Nature 1991 351 652 654 95 10.1038/351652a0 1904993

[B44] Holloway AK Lawniczak MK Mezey JG Begun DJ Jones CD Adaptive gene expression divergence inferred from population genomics PLoS Genet 2007 3 2007 2013 95 10.1371/journal.pgen.0030187 17967066 PMC2042001

[B45] Jagadeeshan S Singh RS Rapidly evolving genes of drosophila: Differing levels of selective pressure in testis, ovary, and head tissues between sibling species Mol Biol Evol 2005 22 1793 1801 95 10.1093/molbev/msi175 15917496

[B46] Kulathinal RJ Singh RS The nature of genetic variation in sex and reproduction-related genes among sibling species of the drosophila melanogaster complex Genetica 2004 120 245 252 95 10.1023/B:GENE.0000017645.84748.dd 15088662

[B47] Begun DJ Whitley P Todd BL Waldrip-Dail HM Clark AG Molecular population genetics of male accessory gland proteins in drosophila Genetics 2000 156 1879 1888 11102381 10.1093/genetics/156.4.1879 PMC1461396

[B48] Lazzaro BP Elevated polymorphism and divergence in the class C scavenger receptors of drosophila melanogaster and D. simulans Genetics 2005 169 2023 2034 10.1534/genetics.104.034249 15716507 PMC1449580

[B49] Jiggins FM Kim KW The evolution of antifungal peptides in drosophila Genetics 2005 171 1847 1859 10.1534/genetics.105.045435 16157672 PMC1456132

[B50] Schlenke TA Begun DJ Natural selection drives drosophila immune system evolution Genetics 2003 164 1471 1480 12930753 10.1093/genetics/164.4.1471 PMC1462669

[B51] Pal C Papp B Hurst LD Highly expressed genes in yeast evolve slowly Genetics 2001 158 927 931 11430355 10.1093/genetics/158.2.927 PMC1461684

[B52] Subramanian S Kumar S Gene expression intensity shapes evolutionary rates of the proteins encoded by the vertebrate genome Genetics 2004 168 373 381 10.1534/genetics.104.028944 15454550 PMC1448110

[B53] Zhang L Li WH Mammalian housekeeping genes evolve more slowly than tissue-specific genes Mol Biol Evol 2004 21 236 239 10.1093/molbev/msh010 14595094

[B54] Gentleman RC V C Bates DM Bolstad B Dettling M Dudoit S Ellis B Gautier L Ge YC Gentry J Hornik K Hothorn T Huber W Iacus S Irizarry R Leisch F Li C Maechler M Rossini AJ Sawitzki G Smith C Smyth G Tierney L Yang JYH Zhang JH Bioconductor: Open software development for computational biology and bioinformatics Genome Biology 2004 5 10.1186/gb-2004-5-10-r80 PMC545600 15461798

[B55] Lemeunier F Ashburner MA Relationships within the melanogaster species subgroup of the genus drosophila (sophophora). II. phylogenetic relationships between six species based upon polytene chromosome banding sequences Proc R Soc Lond B Biol Sci 1976 193 275 294 6967 10.1098/rspb.1976.0046

[B56] Nelson CE Hersh BM Carroll SB The regulatory content of intergenic DNA shapes genome architecture Genome Biol 2004 5 4 R25 10.1186/gb-2004-5-4-r25 15059258 PMC395784

[B57] Dennis G Jr Sherman BT Hosack DA Yang J Gao W Lane HC Lempicki RA DAVID: Database for annotation, visualization, and integrated discovery Genome Biol 2003 4 P3 10.1186/gb-2003-4-5-p3 12734009 PMC193660

