# Supplementary material for: Coordinated evolution of co-expressed gene clusters in the Drosophila transcriptome
Source: BMC Evol Biol. 2008 Jan 7;8:2. doi: 10.1186/1471-2148-8-2 (PMC2266709; doi:10.1186/1471-2148-8-2)
Supplement: Additional File 1 — MA plots. Representative MA plots comparing array results within D. simulans and across species. [file 1471-2148-8-2-S1.ppt]

## Slide 1
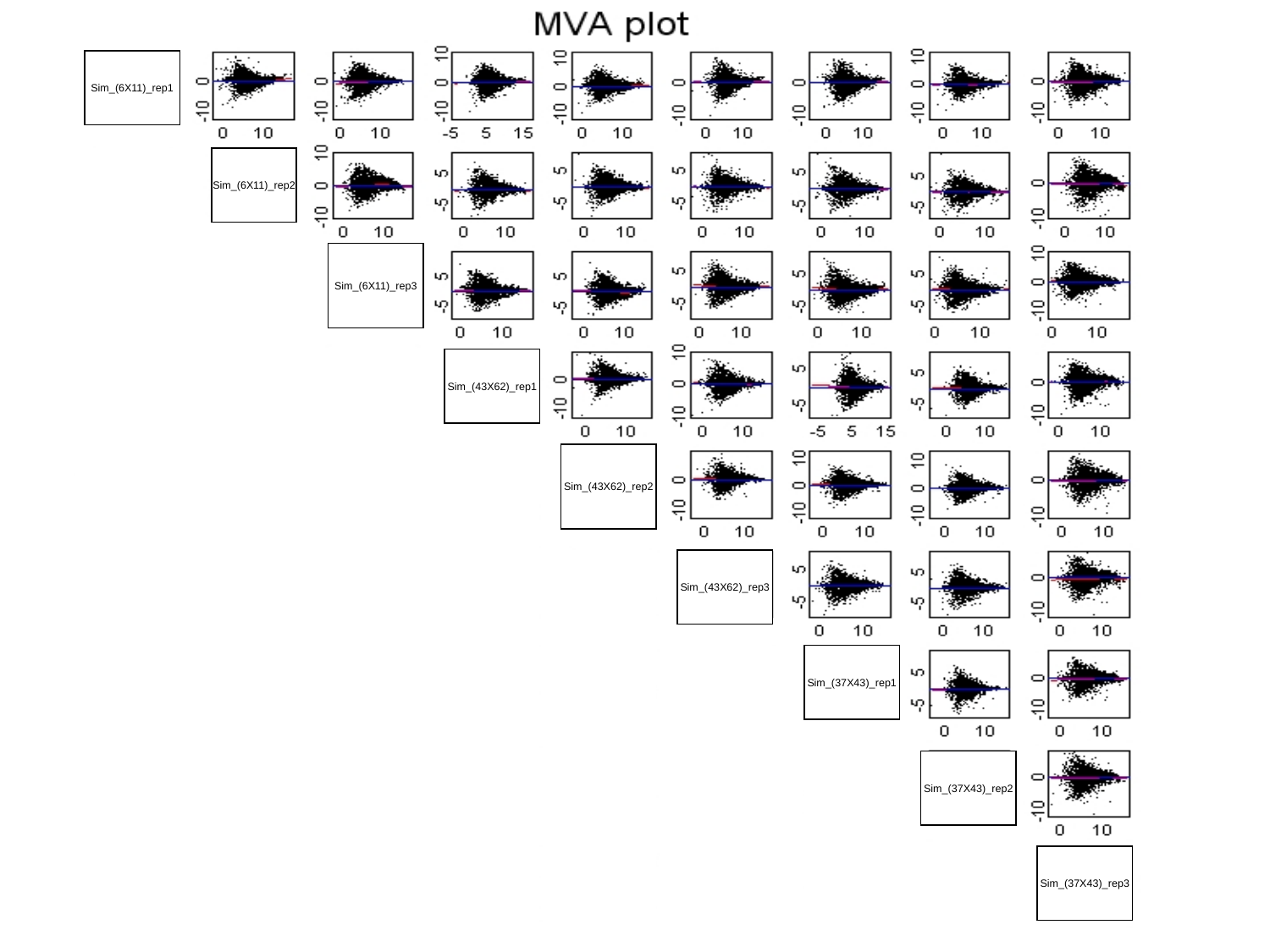

Sim_(6X11)_rep1
Sim_(6X11)_rep2
Sim_(6X11)_rep3
Sim_(43X62)_rep1
Sim_(43X62)_rep2
Sim_(43X62)_rep3
Sim_(37X43)_rep1
Sim_(37X43)_rep2
Sim_(37X43)_rep3

## Slide 2
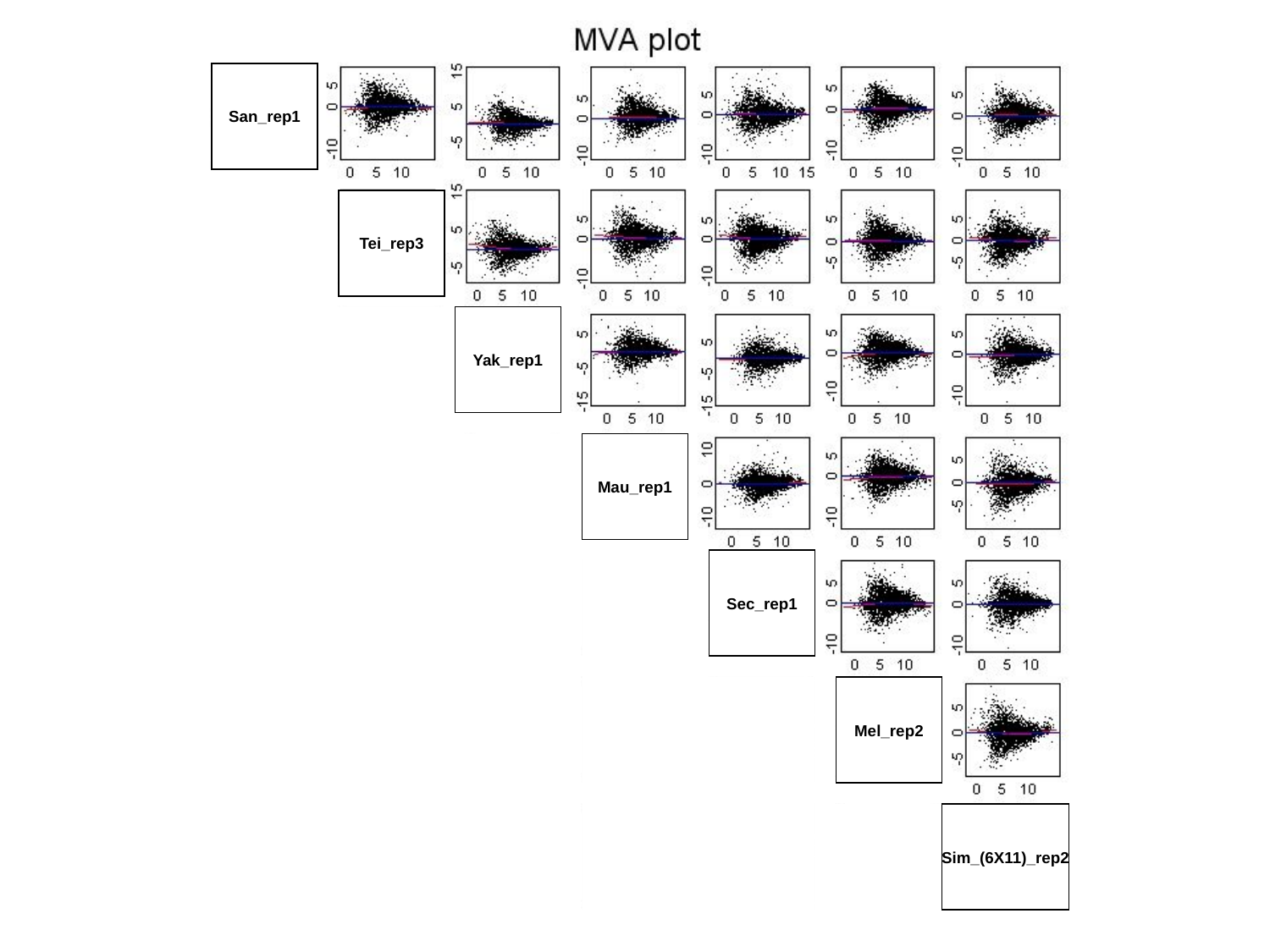

San_rep1
Tei_rep3
Yak_rep1
Mau_rep1
Sec_rep1
Mel_rep2
Sim_(6X11)_rep2
